# Challenges for exploiting nanomagnet properties on surfaces

**DOI:** 10.1038/s42004-024-01183-6

**Published:** 2024-05-01

**Authors:** Guillem Gabarró-Riera, E. Carolina Sañudo

**Affiliations:** 1 Institut de Nanociència i Nanotecnologia, Universitat de Barcelona IN2UB, C/Martí i Franqués 1-11, 08028 Barcelona, Spain; 2 Departament de Química Inorgànica i Orgànica, Universitat de Barcelona, C/Martí i Franqués 1-11, 08028 Barcelona, Spain

**Keywords:** Coordination chemistry, Molecular self-assembly, Information storage, Quantum information, Surface assembly

## Abstract

Molecular complexes with single-molecule magnet (SMM) or qubit properties, commonly called molecular nanomagnets, are great candidates for information storage or quantum information processing technologies. However, the implementation of molecular nanomagnets in devices for the above-mentioned applications requires controlled surface deposition and addressing the nanomagnets' properties on the surface. This Perspectives paper gives a brief overview of molecular properties on a surface relevant for magnetic molecules and how they are affected when the molecules interact with a surface; then, we focus on systems of increasing complexity, where the relevant SMMs and qubit properties have been observed for the molecules deposited on surfaces; finally, future perspectives, including possible ways of overcoming the problems encountered so far are discussed.

## Abstract (web summary)

Molecular complexes with single-molecule magnet or qubit properties are great candidates for quantum information storage and processing, however, device implementation requires controlled surface deposition and property retention, which is a challenge. This Perspective gives a brief overview of molecular properties on a surface relevant for magnetic molecules and how they are affected by surface deposition, pointing out possible ways of overcoming the problems encountered so far.

## Introduction

In the early 1990’s researchers discovered that molecules were able to function as magnets when studying the complex [Mn_12_O_12_(MeCOO)_16_(H_2_O)_4_] (Mn_12_)^1^ This was a huge breakthrough since it showed that molecules could be used to store information by using the spin up and spin down orientations of the molecular spin as binary bits 0 and 1. These molecules were called single-molecule magnets, SMMs. The main caveat here was the operational temperature, which was in the liquid helium regime (below 4 K), and the integration of the molecules into a functional information storage device. The race to discover better, more efficient SMMs with higher operational temperatures has led to chemists preparing large numbers of compounds, with transition metals (3d)^2,3^ with lanthanoid ions (4f)^4^ or combining both transition metals and lanthanoid ions (3d-4f complexes)^5^ The benchmark sign for SMMs has been the highest temperature at which hysteresis of the magnetization vs. field is observed since this is the temperature at which the SMM can be magnetized and thus used to store information, with a bistable spin up and spin down state. The best results so far were obtained in 2021 with an organometallic Dy(III) analog to ferrocene^6^ that showed the SMM property just above liquid nitrogen temperature, but the organometallic SMMs have a great weak point: their extreme instability towards oxygen, humidity, and temperature.

The studies of SMMs and other molecular nanomagnets have led to other great discoveries, one of the main advances has been the demonstration of quantum effects in macroscopic systems. The energy levels of molecules are quantized, and the hysteresis loops of magnetization vs. field of SMMs showed steps^7–12^ These steps correspond to quantum tunneling of the magnetization at specific magnetic fields. The ability to manipulate quantum states in macroscopic molecular samples has driven a new area of interest for chemists and physicists alike: the synthesis and study of molecular quantum bits (qubits) and qudits^13–16^ A qubit is a bistable system, in which the two states can coexist in a quantum superposition of states, in contrast to a classical bit that can exist in two defined states. Qubits are the basic units for quantum information processing and encoding in a quantum computer. In a qudit, there are more than two states. Molecules have quantized energy states that can be initialized, manipulated, and read-out and can thus be exploited for the development of quantum technologies like quantum computing^17^ and quantum sensing^18^ Scientists have raced to prepare the best qubits, using the spin-lattice (T_1_) and spin-spin (T_2_ or T_m_, in this paper we will use T_2_) relaxation times as benchmark values to compare the best performers. T_2_ is also called phase memory time, and it is understood as a measurement of the qubits' coherence time, the time the superposition of quantum states persists, and quantum operations can be performed. Typical T_2_ times for molecular qubits are in the microseconds range for temperatures below 77 K. The loss of quantum information is called decoherence, and it is caused by the qubit interacting with the environment. This is very relevant in the solid state. The main sources of decoherence are lattice vibrations or phonons, and magnetic fields. The vibrational phonons can be controlled by low temperatures or rigid ligands. The magnetic interaction with other nearby spin or nuclear spins can be minimized, for example by dilution in a diamagnetic matrix or by avoiding nuclear spins near the spin qubit^17^ By manipulating chemical synthesis, values for T_1_ and T_2_ long enough that allow manipulation of the quantum system have been achieved. Thus, this means molecular qubits can be exploited in quantum computing devices.

Thus, it has been clearly established that molecular complexes are great candidates for information storage or quantum information processing technologies due to the advantages of chemical synthesis: the molecules are homogenous and reproducible in size and shape, and their properties can be fine-tuned by chemical design. Proposals for the use of magnetic molecules on devices for spintronics or quantum technologies have been many. However, the realization of the proposed molecular-based devices is still not achieved. It is clear that the integration of molecules in devices is a great challenge, and it requires ways to organize molecules on surfaces, and on conductive and magnetic substrates. Additionally, new techniques to address single molecules or ensembles of molecules will have to be devised. A review of proposed device architectures for molecular qubits was recently published^19^ This is a multi-disciplinary challenge; however, the first step relies on chemists and physicists attempting surface deposition of molecular systems. An extensive literature review that covers deposition methods for nanomagnets, types of surfaces, and characterization techniques was published in 2023^20^ Since Mn_12_ SMMs were envisioned as possible molecular bits for information storage, scientists have been attempting the surface organization and the demonstration that the SMM properties were retained on a surface. This proved to be a rocky road, with many trial and error experiments reported. In conclusion, Mn_12_ was too reactive or unstable in the experimental conditions. For a while it seemed that retaining SMM properties on a surface was a huge issue, until other more robust molecules were studied. Fe_4_, which was a much poorer SMM than Mn_12_ with lower hysteresis temperature^21^ was successfully deposited intact on Au(111). The hysteresis of magnetization vs. field for Fe_4_ on a surface was confirmed at 0.70 K^22^ After this turning point, several other SMMs and qubit prototypes have been deposited on surfaces^20^ The latest advances on surface deposition of complex molecules bring the proposals of using molecular systems on devices for information storage and quantum information processing closer to realization. Nevertheless, measuring their SMMs or qubit properties while on a surface is still a challenging task. Advances in studies of the properties of single molecules or small ordered ensembles of molecules on surfaces by scanning tunneling microscopy (STM) and X-ray circular magnetic dichroism (XMCD)^22^ have been critical to ascertain that the promise of retention of molecular properties on a surface is grounded in facts. The challenge remains that, when molecules have complicated structures, reaction with the substrate as well as decomposition or changes in the coordination sphere happening during deposition, might greatly affect or completely destroy their performance.

Once on a substrate, surface-molecule interactions can affect or modify^23–25^ the properties we expect to exploit. It is well known that heterostructures of magnetic and non-magnetic materials can result in changes in the magnetic properties of the materials due to the spinterface^26^ For iron oxide nanoparticles (IONPs), the magnetic molecules on the surface can have a strong effect on the magnetic properties of the nanoparticles, so the molecular layer affects the substrate^27,28^

It is now clear that substrate phonons and the electron cloud of metallic substrates can couple with the magnetic molecules and affect the nanomagnets properties: to solve this, intermediate or buffer layers have been used with success, for example in the case of [Tb(Pc)_2_] SMM, (Pc = phthalocyanine ligand), from now on TbPc^29^ What happens with other compounds, with more complex structures than the TbPc SMM? There are several examples that show that a buffer layer (that can be organic or inorganic in nature) can be used effectively to ensure a weak molecule-surface interaction^28,30–32^ In fact, the organic buffer layer can also be used to graft the molecule on a substrate^33–35^

**Fig. 1:**
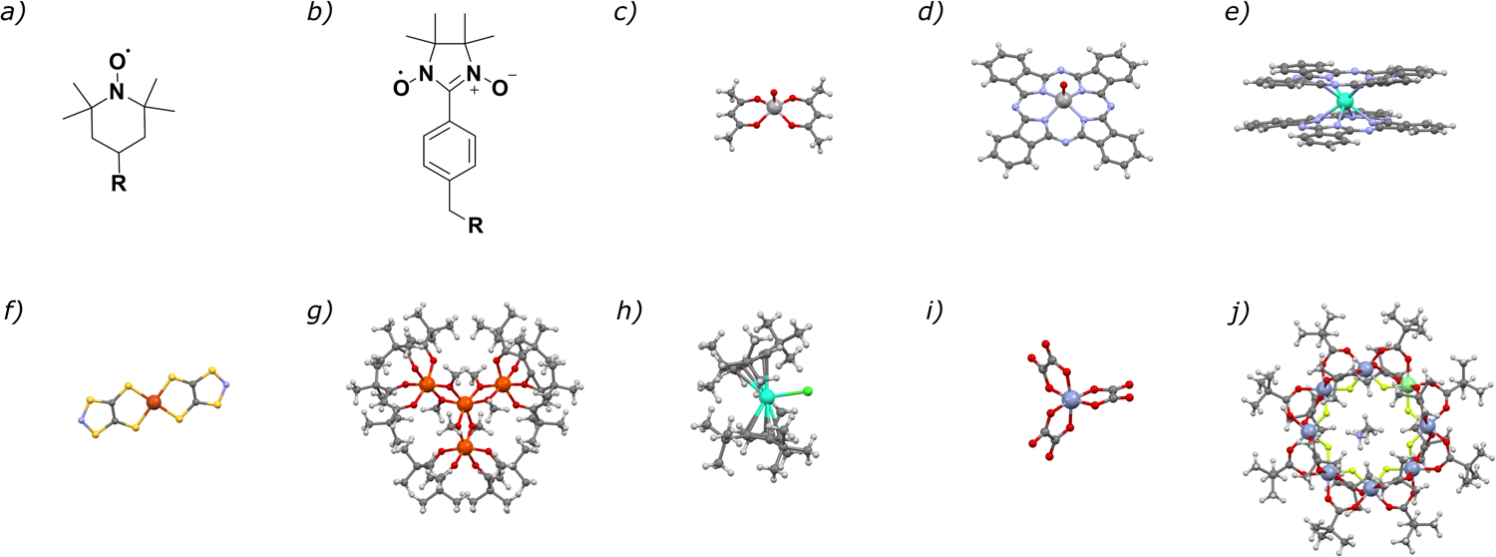
Structures of several qubits and SMMs. Structure of several organic and coordination complexes with qubit or SMM properties. **a** TEMPO radical ((4-prop-argyl-2,2,6,6-tetramethylpiperidine-1-oxyl)^34^ **b** nitronylnitroxideradical^120^ **c** VOacac, vanadyl bisacetylacetonato complex^121^ **d** VOPc, vanadyl phthalocyanine^122^ **e** TbPc, [Tb(Pc)_2_]^123^ **f** Cudtt, [Cu(dttt)_2_], where dttt- = 1,3,2-dithiazole-4-thione-5-thiolate^81^ **g** Fe_4_, [Fe_4_(MeO)_6_(dpm)_6_], where dpm = dipivaloylmethane^21^ **h** [Dy(Cp^ttt^)_2_Cl], where Cp^ttt^ = =1,2,4-tri(*tert*-butyl)cyclopentadienide)^36^ **i** chromium oxalate^124^ **j** Cr_7_Ni, (Me_2_NH_2_)[Cr_7_NiF_8_(^t^BuCOO)_16_]^125^.

Figure 1 shows the structure of SMMs and qubits that have been deposited on surfaces. The best SMMs known to date, the lanthanoid metallocenes, are extremely air sensitive so their surface deposition presents some outstanding challenges^6,36,37^ Many of these qubits have more complex structures than simple phthalocyanine complexes. Even simple qubits like the M(III) trisoxalates (M = Cr, Ru, Fe) are affected by changes in the second coordination sphere, thus making their use on surfaces quite complex^38–40^

Molecular properties of qubits and SMMs are necessary to integrate these molecules in functional technologies for quantum information processing in a quantum computer or for information storage in hard drives, however, molecules need to retain their properties while they are part of an electronic device, most likely deposited on a surface. Figure 1 shows the structure of some of the most relevant compounds that have been deposited on substrates, which will be discussed in the coming sections of this article.

To summarize, this Perspectives paper gives a brief overview of molecular properties on a surface relevant to magnetic molecules and how they are affected when the molecules interact with a surface; then, we focus on systems of increasing complexity, where the relevant magnetic properties have been observed for SMMs and qubits deposited on surfaces; finally, a future perspective, including possible ways of overcoming the problems encountered so far are discussed.

## Molecular properties on a surface

SMMs and molecular qubits hold great promises for their application on devices^41–45^ Molecular systems have great advantages over classic inorganic solids, but after more than thirty years of researching new SMMs, we still cannot find a true application being reported. In a sense, this is mainly due to the overspecialization of science: synthetic chemists prepare new complexes with better properties, but it seems hard to integrate these compounds into functional devices without the collaboration of physicists and engineers. Over the last few years many research efforts have been devoted to showing that SMMs could be deposited on surfaces effectively and without affecting the molecular properties, and thus, SMMs could actually be integrated into a device as surface-deposited molecules. The main property of SMMs that should be retained on a device is the operational temperature, that is the temperature below which the SMM can retain the magnetization after removal of an applied magnetic field and hysteresis of the magnetization is observed. Chemists very often report the highest temperature at which an out-of-phase AC signal is observed for an SMM. However, it is often the case that hysteresis appears at temperatures that are lower than the peak at the out-of-phase AC signal. In this work, we will only refer to operational temperature as the highest temperature at which hysteresis is observed. Furthermore, the hysteresis should have remanence, which is often reduced in the bulk due to strong quantum tunnelling of the magnetization. To measure magnetic hysteresis of molecular thin films, the technique used is XMCD in a synchrotron beamline. Magnetic hysteresis loops at different temperatures and sample orientations can be obtained by monitoring the most intense XMCD signal as a function of the applied magnetic field. This is, for example, the M_5_ edge signal for Tb in a Tb SMM. Most synchrotron beamlines for XMCD are equipped with 5 T or 7 T magnets and liquid helium cryostats. The magnetic field sweep rate depends on the synchrotron beamline, but a typical value is ca. 8 mT s^−1^.

Table 1 compares the relevant properties for SMMs (hysteresis temperature) and qubits (T_1_ and/or T_2_) between bulk materials and surface-deposited molecules. Hysteresis of the magnetization was demonstrated for Fe_4_ on Au(111)^22^ and later on for TbPc SMM on Au(111)^46^ highly oriented pyrolytic graphite (HOPG)^47^ and MgO/Ag^29^ Recently, hysteresis of the magnetization similar to that of the bulk was reported for a 2D array of endohedral fullerenes^48^ A graphene layer was placed in this example between the metal surface and the SMMs. Except for the MgO/Ag surface, all hysteresis loops observed were butterfly-like hysteresis, without any relevant remanence, due to fast zero-field quantum tunneling. Thus, we know that SMMs can be used as such while deposited on surfaces but still most of the time the properties are below the benchmark known for bulk samples. In one of the examples in Table 1, a synergistic effect between surface and molecule gives rise to an enhanced hysteresis loop for the SMM-nanoparticle system^28^ However, this effect precluded control of the magnetization of the SMM molecules in a potential information storage device, as shown using XMCD. New phenomena can be obtained from synergistic molecule-surface effects, but these are not the aim of this paper.

**Table 1:** Comparison of properties for bulk and surface deposited molecules. For SMMs, hysteresis temperatures are compared. For qubits, relaxation times are compared

SMM	T Hysteresis (bulk)	Surface	Hysteresis on surface	T Hysteresis (on-surface)	Reference
TbPc	7 K	MgO/Ag(100)	Yes	3 K	^29^
		Ag(100)	No	–	
		HOPG	Yes	7 K	^47^
		Au(111)	Yes	15 K (200 nm film) 4.5 K (monolayer)	^46^
Fe_4_	< 1 K	Au(111)	Yes	0.9 K	^72^
		Au(111)	Yes	0.7 K	^22^
		Au(111)	Yes	0.65 K	^119^
Dy_12_	< 1 K	IONPs	Yes	30 K*	^28^
(Cp)^ttt^_2_DyCl	< 2 K	SiO2 particles	Yes	8 K	^39^
Dy_2_@C_80_(CH_2_Ph)	20 K	graphene/Ir(111)	Yes	20 K	^48^
* synergistic effect with IONP, hysteresis of pristine IONP at 10 K					
**Qubit**	**T**_**1**_ **and/or T**_**2**_ **Bulk**	**Surface**	**T**_**1**_ **and/or T**_**2**_ **on-surface**		**Reference**
[Cu(dttt)_2_]	T_2_ = 2.3 µs	Kapton	No		^81^
TEMPO radical	T_1_,slow = 57 ms	Au/Kapton	T_1_ = =9.2 ms		^34^
	T_1_,fast = 14.4 ms				
	T_2_ = 3.23 µs		T_2_ = =13.53 µs		

**Fig. 2:**
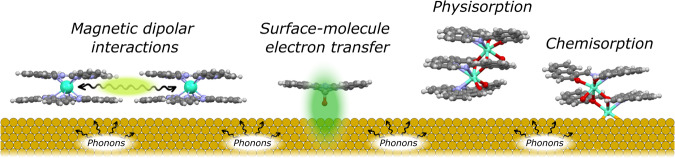
Illustration of most relevant molecule-surface interactions. Most relevant interactions that affect the properties of SMMs and qubits while deposited on a substrate. The results reported for the TbPc SMM emphasize the importance of the environment for SMMs: on a surface, one must consider the dipolar interactions between closely packed molecules in 2 dimensions and the interaction with the surface. This is not unique for LnPc SMMs, in fact, for bulk SMMs magnetic dilution has been a clear strategy to improve SMM properties^126^ In this way, in magnetically diluted systems one reduces dipolar interactions between nearest neighbors. In order to consider molecule-surface interactions, the surfaces can be classified as involving localized electrons (covalent or ionic structures that can be treated as dielectrics) or delocalized electrons (metals and semiconductors, for which the band structure of the solid must be considered). For chemisorbed molecules on surfaces (Fig. 2d), that is when there is electronic rearrangement and bond formation between the molecule and the surface, one must very carefully consider the coordination environment of the paramagnetic ions in the nanomagnet, whether this is an SMM or a qubit candidate. Large changes in the coordination environment inevitably lead to modifications in the crystal field and changes in hysteretic properties or in the T_1_ and T_2_ relaxation times for qubits. Most strategies to avoid changes in the relevant magnetic properties rely on using long organic tethers to graft or chemisorb the nanomagnet on the surface. This introduces a large spatial separation between the surface and the SMM or qubit that avoids strong hybridization. The chemical design of the tether groups is a key factor when attempting chemisorption.

Our aim is to understand how molecular properties like magnetization hysteresis or T_1_ and T_2_ are affected by the molecule-surface interaction. How are the blocking temperature and remanence affected by the surface deposition? Fig. 2 shows the most relevant molecule-surface interactions.

Much less work has been devoted to depositing qubits on surfaces, and to in fact ascertaining that the T_1_ and T_2_ relaxation times characteristic of the molecular species are reproduced when the molecules are deposited on a surface. Most of the time, the T_1_ and T_2_ times reported on papers have been obtained using electron paramagnetic resonance (EPR) on isolated qubits in frozen solutions or magnetically diluted powders. In order to measure the relaxation times, dipolar interactions between qubits must be minimized, since magnetic interactions lead to efficient relaxation pathways. This is achieved in solid state by doping the active qubit in a diamagnetic matrix. In solution, dipolar interactions are minimized by dilution in a solvent. A diamagnetic compound that is isostructural to the known qubit system must be prepared for magnetic dilution in solid state. For lanthanoid complexes, this can be achieved by preparing La(III), Y(III), or Lu(III) analogues of the Ln-containing qubit. For transition metals, diamagnetic analogues with Zn(II) or Ga(III) can sometimes be prepared, but this must be studied case by case and is not straight-forward. In most cases, diamagnetic analogues can be prepared, but the detailed structural and chemical characterization is necessary. On the other hand, as mentioned before, EPR is most usually measured in frozen solutions by using organic solvents that form glasses upon freezing. This is done by diluting the qubits in a suitable solvent and cooling them down to a temperature where the solvent provides a glassy matrix. The main problem in the frozen solution method is that the solubility of the desired qubit must be correctly assessed and that in some cases the structure might suffer subtle changes when the crystals are dissolved. For example, a complex [MLS_2_] that contains terminal solvent (S) ligands, such as pyridine can be dissolved in THF. In solution, the terminal solvent ligands will be in fast exchange with the solvent (S') forming [MLSS'] or [MLS'_2_]. This means that the coordination sphere of the metal will most likely be different from that revealed by the crystal structure, this in turn means the crystal field will be different^49^ So, in the frozen solution it is not easy to establish the nature of the species whose T_1_ and T_2_ are being recorded. Of course, this only applies to coordination complexes that contain terminal solvents, or ligands that can easily be exchanged by a solvent with a donor atom. For example, trischelate-metal qubits of general formula [ML_3_] where L is a chelate ligand such as oxalate^50^ or dithiolate do not present this problem, since they will remain intact in solution^51^

Thus, the challenge of placing the qubits on a surface to address their magnetic properties has added difficulties, mainly related to how the T_1_ and T_2_ relaxation times are reported for the qubit and how they are measured by EPR. On the surface subtle changes in the crystal field might happen. Furthermore, EPR requires a minimum number of spins in order to detect the signal: this is of the order of 10^14^ spins. A close-packed monolayer can contain of the order of 10^13^ molecules/cm^2^ so in principle it should be possible to detect the monolayer by EPR. The standard EPR cavity is not suitable to collect the EPR spectrum on a flat sample. In most commercial EPR spectrometers, the sample is placed inside a quartz tube with a diameter of 4  mm. The sample is placed in the tube as a powder or a solution. There are specific setups for single crystals and thin films. Recent reports on new EPR resonator design that concentrate the field strength in a 2D area showed great performance for thin-film and monolayer samples^52^ Further advances in this technology will indeed be very interesting for the study of the magnetic properties of molecular monolayers or thin layers. The resonators used for biological samples^53^ or for single crystals can be used to collect data on flat surfaces. For this, the main elements are the concentration of spins in the monolayer and the surface material. The sensitivity of EPR was briefly commented on above. However, the nature of the surface material must also be considered. Microwave transparent materials must be used, this rules out the most common substrates for surface deposition studies: silicon wafers, and Au or Cu single crystals. Materials like MgO, SiO_2_ or Al_2_O_3_ can be used. Additionally, thin (less than 100  nm) layers of Au or other metals are also transparent to microwaves. All these materials can contain impurities that can give signals detectable by EPR and that can be stronger than the signal from the molecular qubits deposited on the surface^54,55^ The pulsed experiments to detect T_1_ and T_2_ should be possible providing that the dipolar interactions in the monolayer are less relevant than in the bulk solid. Magnetic dilution in the monolayer to avoid dipolar interactions that may cause decoherence is possible if a suitable diamagnetic analogue exists, but of course this adds extra complications to the system since the number of spins on the surface might then be too low for detection.

Advances in STM have led researchers to explore ESR (electron spin resonance, used in this field instead of the term EPR) on single molecules or even single atoms on a surface using STM^56,57^ In this way, the cryogenic initialization and universal multi-qubit operations and multi-qubit detection using electron spin qubits on surfaces have been demonstrated^58^ ESR-STM was used in 2022 to detect the ESR signal of single FePc-molecules formed upon nanofabrication on a MgO/Ag(100) surface^59^ The main drawback of ESR-STM is that it is limited to species amenable to nanofabrication and scaling to large number of qubits is a great challenge. Additionally, an important drawback for ESR-STM advocates to address is the decoupling of the molecules from the surface. So far, molecules are decoupled from a surface by placing an intermediate layer that avoids hybridization of the molecular orbitals with the surface. The intermediate layers are self-assembled monolayers (SAMs) of organic molecules, with ref. ^34^ or without^28,30,31,33^ long tethered organic groups. These two cases complicate the use of ESR-STM. However, few monolayers of MgO can be used as demonstrated for TbPc^29^ and later in an ESR-STM study of FePc^59^ The exploration of MgO as a suitable substrate for qubit and SMM deposition might lead in the coming years to advances that might help researchers overcome this drawback.

## Observation of magnetic hysteresis for SMMs on surfaces

Most of the SMMs studied on surfaces to date have very low blocking temperatures, below or around liquid helium (4 K). The SMM selection is done on the basis of availability, or the possibility of sublimation of the SMM in ultra-high vacuum (UHV) since this is the way to achieve the cleanest submonolayers or isolated molecules on a substrate.

In 2016, the observation of a hysteresis loop with strong remanence for TbPc on MgO/Ag(100) was reported. The layer of a non-magnetic insulator (MgO) between the molecules and the silver substrate allowed to control the tunnelling rate^29^ The importance of considering the disruptive effect of the surface on the nanomagnet’s properties is truly highlighted in this example, considering that butterfly hysteresis loops had already been obtained for TbPc on other substrates like Au(111)^46^ or HOPG^47^ For the first time, the surface adsorbed molecule outperforms the bulk. The effect is attributed to the efficient protection from electron scattering and weak molecule-surface hybridization by the MgO/Ag(100) substrate^60^

Spin-phonon coupling is also important for bulk SMMs. A control of spin-phonon coupling is key to observing hysteresis at high temperatures for dysprosium metallocene SMMs^6,37,61,62^ These SMMs hold the record of displaying hysteresis at 80 K, just above liquid nitrogen cryogenic temperature^6,36^ This was a great milestone, and signaled that SMMs could be in fact integrated in devices that would not need liquid helium. However, the manipulation of these species is complicated since they are extremely unstable to air, moisture, and temperature. Using amorphous silica as a surface, Dy-cyclopentadienyl fragments (with various substituents) have been chemisorbed using the chemistry of the silica surface^38,40^ or by introducing Lewis acid sites on the silica surface^39^ The former attempts did not result in hysteresis temperatures similar to the parent SMMs, but did help build the knowledge needed to improve the surface deposition of dysprosium metallocene SMMs. In the last example, a poor SMM of the metallocene-type [Dy(cp^ttt^)_2_Cl] (see structure in Fig. 1h) is chemisorbed on Aluminum Lewis acid sites on silica. The chloride ligand bridges the SMM to the aluminum sites, resulting in a modification of the coordination environment on the Dy(III) ion. The bulk SMM does not show hysteresis above 2 K^36,63^ however, the chemisorbed species shows hysteresis of the magnetization opening up to 8 K. Clearly, in this case, the isolation of the SMMs on the silica surface along with the tuned coordination environment of the SMM results in an improvement of the magnetic properties. This is an extremely interesting result that further motivates researchers in the field to pursue the surface deposition of SMMs. Furthermore, this is a unique example of surface deposition of dysprosium metallocene SMMs on amorphous silica, that could probably be extended to flat surfaces. Nanoparticles (NPs) are great model systems to probe magnetic properties (they can be centrifuged or precipitated and placed into a SQUID magnetometer, and EPR tube...) but they are not easily integrated into an electrode or a heterostructure, in particular if we pretend to control/know the orientation of the qubits or SMMs with respect to an applied magnetic or electric field. It should be noted that NPs are spherical as a rough approximation or many-faced polyhedra (cube, truncated cube, etc.). With a flat surface, we refer to a surface where there is a clear orientation with respect to an imaginary xyz axis set (xy is the plane of the surface, z, perpendicular to the plane). For a crystalline NP, each facet is xy. So far, the hysteresis temperature and remanence of TbPc and a dysprosium metallocene SMM have been improved by surface deposition. Other examples of hysteresis enhancement of SMMs deposited on magnetic surfaces suggest a synergy between the surface and the surface-deposited SMM but the observed hysteresis could not be attributed to the SMM^28^

## Qubit relaxation times on surfaces: Organic radicals and coordination complexes

Simple, stable organic radicals are a potential group of qubits. TEMPO and nitronylnitroxide radicals are shown in Fig. 1a, b. The spin state is a pure one electron S = 1/2 stable through a wide temperature range. This is an advantage over complex coordination complexes that have an S = 1/2 spin ground state that is achieved by magnetic coupling of two or more metal ions, thus the S = 1/2 is only a robust spin state that can be exploited for quantum computing as long as the temperature is low enough so that excited states are not thermally populated or Zeeman splitting does not result in level crossings. The extent of this stable range for S = 1/2 for a magnetically coupled system largely depends on the strength of the exchange coupling constant J, and usually limits operational temperatures of coordination complexes as qubits to the liquid helium temperature regime. Organic radicals can be robust in a wide range of conditions, they can be synthetically modified to engineer them to be chemisorbed on a substrate^34^ or to form part of a ligand in a metal-organic framework (MOF)^64^ The spin-lattice and spin-spin relaxation times (T_1_ and T_2_) of several organic radicals have been determined by pulsed EPR in frozen solutions. Nuclear spins within 12  Å of the electron spin contribute to decoherence, and protons as far away as 8 Å from the spin are the strongest contributors^65^ The observed T_1_ and T_2_ relaxation times for organic radicals are relatively long at temperatures achievable with liquid nitrogen, thus, they are excellent candidates for qubits in quantum computing^65–67^

Organic radicals have been deposited as monolayers or as part of alkane-thiol SAMs, for a variety of applications^68–70^ In 2007, the CW-EPR of a SAM of organic radicals on Au(111) was reported^71^ These reports established the mobility of radicals on a long organic tether covalently bonded to a surface and highlight the importance of the deposition method. Theoretical studies also conclude that for efficient quantum coherence as needed for quantum computing applications, the qubit must be decoupled from the surface. The hybridization of the radical with the surface must be avoided, thus the necessity of a long tether group. As with complex molecules such as the SMMs described in the previous section, organic tethers can be used to create a physical separation between molecule and surface that is enough to ensure a decoupling^72^ but the long tether introduces the mobility that leads to domains of radicals with different orientations on the surface.

Only very recently the T_2_ of layers of organic radicals chemisorbed on gold were reported^34,73^ The first example was reported in 2021, nitronyl-nitroxide radicals were chemisorbed on Au(111) and pulsed EPR was used to measure T_2_ of the nano-structure at 80 K. The T_2_ time obtained for isolated chemisorbed radicals was similar to the value measured on frozen solutions^73^ In this work, several domains of organic radicals directly chemisorbed on Au(111) are reported even though they are not quantified, and the observed long coherence times are attributed only to disordered, low density packed radical domains on the Au(111) surface.

In a more recent work, a stable nitroxy radical, TEMPO (2,2,6,6-Tetramethylpiperidine-1-oxyl) is covalently linked using click-chemistry to a SAM of azide-functionalized alkanethiols on Au(111)^34^ The authors show that there might be some radical mobility due to the alkane tethers of the alkanethiol SAM and detect and interpret the continuous-wave EPR signal for the surface grafted TEMPO radicals based on an exchange coupled model that includes a 1:1 mixture of rigid and quasi-mobile radicals. Furthermore, they are able to detect the spin-spin and spin-lattice relaxation times (T_2_ and T_1_) of the surface-grafted TEMPO radicals using pulsed EPR fitted with a home-built Fabry-Pérot resonator. In this case, the close packing of the qubits in 2D layers leads to substantial magnetic coupling, which according to the authors does not impair the relaxation time of the TEMPO radical. T_1_ is very similar for the surface-grafted TEMPO to the T_1_ obtained for frozen solutions of the TEMPO derivative used in the grafting. Furthermore, T_2_ is also of the same order for surface-grafted TEMPO to the T_2_ measured for a frozen solution of the precursor. This is a surprising result, as it is widely accepted that dipolar interactions are important in closed-packed systems and they are a strong source of decoherence from molecular qubits. As the authors point out, the 2D nature of the SAM of TEMPO radicals limits the decoherence mechanisms present in the spin bath and might explain the similarity in T_1_ and T_2_ with the bulk, but the role of the exchange interactions is still being investigated.

Coordination complexes have been proposed as molecular qubits in two distinct categories: pure S = 1/2 systems such as Cu(II) or vanadyl complexes, or polymetallic complexes with strongly coupled spin ground state S = 1/2. Coordination complexes with spin ground state larger than 1/2 are also interesting for quantum computing as qudits. Unlike qubits, which can exist in a superposition of just two states, qudits can exist in a superposition of more than two states. Thus, a system employing qudits would offer more computational resources and advantages in certain quantum algorithms and applications.

Vanadyl complexes are simple, pure S = 1/2 systems. Several vanadyl compounds have been studied as qubit candidates. Acetylacetonanate (acac) and dipivaloylmethane (dpm) vanadyl complexes (VOacac and VOdpm) are mononuclear species. The dpm analogue, with tert-butyl groups, is ideal for evaporation. VOdpm molecules can be easily sublimated on a surface, submonolayers of VOdpm were deposited on Au(111)^74^ opening up the possibility of electrically addressing the qubits by using a metallic substrate. Vanadyl phthalocyanines (VOPc) have been one of the most studied vanadyl compounds on surfaces since they are also amenable to evaporation. Upon deposition, VOPc molecules adopt two different adsorption geometries: lying flat with the oxygen pointing to the vacuum (oxygen-up) or with the oxygen pointing to the substrate (oxygen-down). On superconducting Pb(111), the interaction with the substrate is almost negligible in the oxygen-up configuration, as opposed to the oxygen-down molecules, which presented a stronger interaction. However, XMCD studies demonstrated that the S = 1/2 ground state was unaffected after deposition, even if the interaction with the substrate was strong^75^ On Si(111) substrates, submonolayers of VOPc were found to favor the oxygen-down configuration, due to the formation of a new bond between the Si atoms and the oxygen from the vanadyl group. On the other hand, less reactive substrates such as Ag(111) or Au(111) favored the oxygen-up configuration^76,77^ On Ni(111), VOPc molecules presented a strong interaction with the metallic substrate, which could be effectively suppressed by adding an intermediate graphene layer^78^ Similar results were reported some years later on Graphene/SiC(0001)^79^ These results point out again the importance of choosing the right substrate to avoid specific interactions that could lead to the loss of the compound’s magnetic properties. CuPc (copper(II) phthalocyanine complex, with S = 1/2) is also a good qubit candidate. In 2013 researchers reported T_1_ and T_2_ of 400  nm thick films of CuPc diluted in H_2_Pc over functionalized kapton^80^ Long T_1_ times obtained show that the simple CuPc molecule holds promise for quantum information processing. Furthermore, even though the system is not a monolayer and a 400  nm film can be considered similar to the bulk, the film is prepared with easily processable materials and resorts to a very smart strategy of isolating the qubits from each other to avoid dipolar interactions by doping CuPc into a free ligand matrix.

Exploiting the idea of nuclear-spin-free environments to improve T_2_, the synthesis and deposition of molecular complexes of hydrogen-free 1,3,2-dithiazole-4-thione-5-thiolate (dttt−) ligands has been studied^81^ A hydrogen-free ligand environment reduces sources of decoherence caused by the nuclear spin of hydrogen atoms. The authors prepared S = 1/2 dtt- Cu(II) complexes (structure shown in Fig. 1f), as well as diamagnetic analogues with square-planar Ni(II). This allowed the preparation of magnetically diluted Cudtt complexes in Nidtt. The Cudtt compound was magnetically characterized as very strong antiferromagnetically coupled 1D coordination polymers. The spin dynamics of the Cu^2+^ (S = 1/2) compound were studied for the bulk and on a magnetically diluted sample of Cudtt on the diamagnetic Nidtt matrix. Magnetic dilution caused only a small effect in T_2_, attributed by the authors to the presence of ^14^N nuclei, even though interaction with the I = 1 of ^14^N had been deemed irrelevant for similar systems^82^ The authors report 20  nm Cudtt layers and the CW-EPR of the thin layers but no T_2_ measurements are mentioned in the paper.

The qubits commonly known as chromium wheels, [R_2_NH_2_][Cr_7_NiF_8_(pivCOO)_16_] or Cr_7_Ni, are exchange coupled systems with an S = 1/2 ground state and a suitable energy spectrum for qubit encoding and manipulation^83^ A suitably isolated S = 1/2 spin ground state is not the only advantage of the Cr_7_Ni qubits. The Cr_7_Ni wheels can be chemically modified in order to construct more complex systems, quantum gates, in which different qubits are linked together in a system that can perform quantum logic operations. The chemical versatility of these complexes makes them ideal candidates for supramolecular assembly of quantum gates, as demonstrated in 2016 for the CNOT quantum gate^84^ Cr_7_Ni complexes can also be specifically designed for surface deposition. The Cr_7_Ni complexes can be chemically tuned^85^ to be easily evaporated, chemisorbed, or physiosorbed on substrates^85–88^ The extensive family of M_7_M' wheels^83,87^ has allowed researchers to completely understand solubility, stability^89^, and magnetic properties^90^ of the complexes with a great amount of detail. The Cr_7_Ni complex has been chemisorbed and physiosorbed on Au(111) and the sub-monolayers characterized in great detail by STM^91–93^ The magnetic properties of Cr_7_Ni wheels on surfaces have been studied by XMCD, showing that the surface deposition by sublimation preserves the magnetic properties of these molecular rings^92^ However, it seems that T_1_ and T_2_ of Cr_7_Ni qubits has not been demonstrated on surface-deposited complexes.

Many complex molecules have been recently proposed as qubits^94–97^ but surface deposition and measurement of the T_1_ and T_2_ is not straightforward in on-surface samples and still has not been explored in full. Furthermore, chemists are designing qubits for ordered surface deposition like vanadyl/Pt paddlewheels that are expected to have one preferred orientation on a surface^98^ In a 2023 paper, Carreta and coworkers propose a blueprint for encoding, initializing, and reading qudits coupled to superconducting resonators to achieve a molecular spin quantum processor^99^ The qudits selected are heterometallic, dinuclear lanthanoid complexes. The objective is coupling the molecular qudits to superconducting resonators adapted to the molecular qudit size and magnetic interactions to achieve strong spin-phonon coupling. The experimental realization requires sparse surface coverage that they suggest can be achieved by evaporation or self-assembly from the solution. With this approach, the authors suggest that surface order is not necessary and that only the molecules from a randomly oriented ensemble that fulfill the operational conditions will be selected. Undoubtedly, the next few years will bring interesting results regarding the surface deposition of qubits and their implementation in functional devices.

## Ordered arrays of qubits and SMMs as promising new materials

MOFs containing SMMs or qubits have been appearing in the literature in growing numbers in the last 20 years. MOFs are ideal, ordered arrays of metal-organic nodes, and these nodes can be chosen to have the desired properties. Ordered arrays of SMMs can be achieved by preparing MOFs that contain nodes that are known to have SMM properties. Dy(III) magnetic MOFs can serve this purpose and several examples have been reported where the ordered array does not act to the detriment of the magnetic properties of the SMM or qubit moiety^100^ The structures of MOFs are complex, and in some cases, the SMM properties can arise from 1D-chains inside the MOF as shown for Dy(III) and Er(III)^101^ The ligands can be designed to maximize separation between SMM nodes, thus introducing magnetic dilution and reducing dipolar interactions, or to constrain the coordination geometry of the magnetic nodes to improve SMM properties^102^ In a different strategy, porous MOFs can be loaded with SMMs on the cavities^103^ Ordered arrays of qubits can also be achieved exploiting MOF chemistry. This is a topic that has not been thoroughly explored, but offers great advantages for the field of quantum sensors and quantum processors that require high structural precision. A clocklike transition for cobalt(II) spins in the MOF [(TCPP)Co_0.07_Zn_0.93_]_3_[Zr_6_O_4_(OH)_4_(H_2_O)_6_]_2_ has been reported, the qubit here is a well-known tetraphenylporphyirin Co(II) complex diluted with Zn(II) that is trapped on the MOF porous cavities^104^ In this system, the measured T_1_ and T_2_ of the atomic clock transition are relatively long (microsenconds) even at high magnetic concentrations.

The term quMOF has been recently coined, and it refers to MOFs that contain magnetic ions that could behave as qubits (or qudits). Thus, quMOFs offer a new way to obtain the three-dimensional organization of spin qubits. The main issue here becomes the control of the 3D order of the qubits in the 3D MOF or quMOF. If the qubits are magnetically diluted to avoid decoherence by dipolar interactions or magnetic coupling all order is lost, as the qubit will be found randomly in the MOF crystal structure, much like an impurity on MgO or a nitrogen vacancy in the diamond. If the qubits are not diluted, it might be impossible to address individual qubits. Gd(III) qudits were assembled in a quMOF and its magnetically diluted analogues were prepared using Y(III)^105^ The authors report T_1_ and T_2_ times comparable to other molecular qubits at low temperatures and Rabi oscillations up to 40  K for the yttrium-Gd magnetically diluted analogue. They also suggest that quantum coherence could be improved by avoiding the physical motions of magnetic nuclei coupled to spin qubits.

**Fig. 3:**
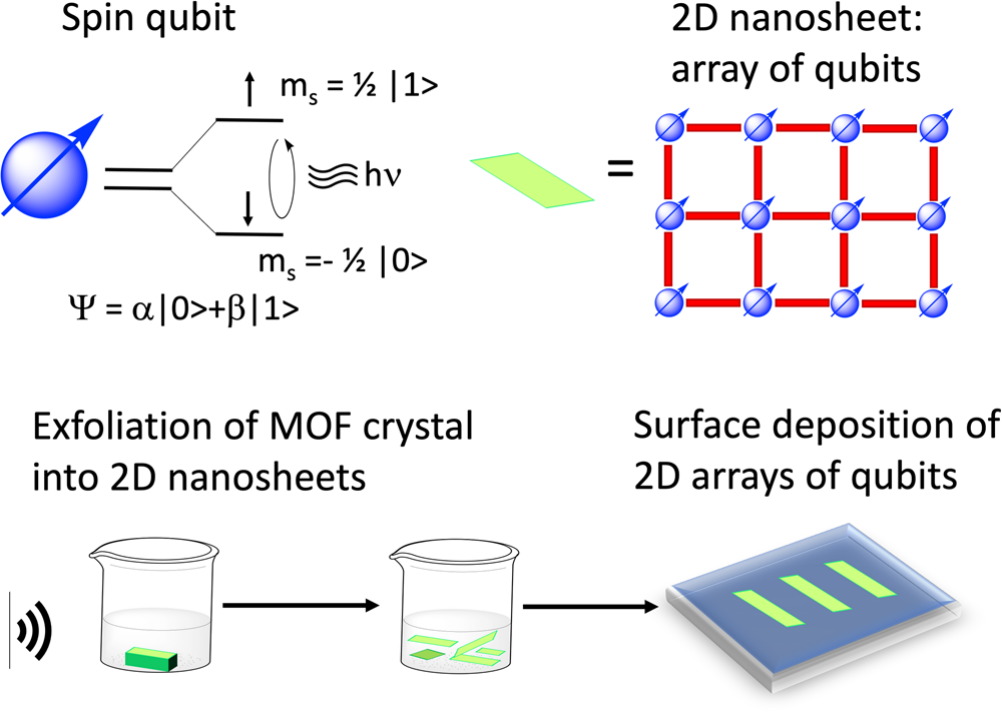
Integration of qubits into nanosheets. Schematic representation of a 2D array of qubits integrated into a nanosheet. The node of the MOF is the qubit and the linker (red rods) can be an organic ligand. The bottom part of the figure illustrates the process of exfoliation and surface deposition^127^. 2D arrays of qubits can be designed to control dipolar interactions and to integrate qubits into devices^127,128^ If the magnetic molecules are organized in a 2D coordination polymer or 2D MOF, intrinsic magnetic dilution becomes possible, as heterometallic complexes can be easily achieved. In this landscape, 2D MOFs offer a variety of possibilities for the implementation of devices using SMMs and qubits^129^ Van der Waals 2D MOFs can also be exploited as 2D arrays of SMMs like [Dy(MeCOO)(PhCOO)_2_]^130^ Furthermore, 2D materials can be easily exfoliated and deposited on surfaces as flakes, as shown for a Tb 2D MOF [Tb(MeCOO)(PhCOO)_2_]^131^ The properties of individual flakes can be studied while on a surface. However, in these cases of physisorbed flakes, there is little control on surface coverage, orientation of the flakes on the substrate, or thickness of the deposited flakes. Also, 2D flakes fall flat on the substrate, so even if the xy orientation of the qubits is not the same for all flakes, the z orientation of each qubit should be the same. In this way ordered arrays of qubits or SMMs are easier to achieve in materials that can be obtained in the form of 2D nanosheets.

2D MOFs offer the possibility of organizing molecular building blocks in 2 dimensions. Figure 3 shows a cartoon of a 2D MOF or 2D nanosheet of qubits, and the exfoliation and surface deposition process that would lead to ordered arrays of qubits. Thus, 2D MOFs are ideal for the integration of molecular nanomagnets into devices since they can be transferred on a surface as monolayers: 2D arrays of magnetic molecules can be fundamental for advances in molecular spintronics applications or devices^20^

Surface growth of MOFs is a topic where important advances have been made that are reflected in literature reviews^106^ and papers that report how the growth of MOFs on a substrate is clearly possible in a controlled manner. Applications are usually centered in areas where MOF chemistry is more focused, like catalysis or CO_2_ capture^107^ Growth can be performed layer-by-layer, thus allowing extreme control on the thickness of the prepared MOF grafted on a surface^108^ The layer-by-layer growth could also be applied to suitable magnetic MOFs or quMOFs. This has already been reported for spin-crossover coordination polymers of the Hoffman clathrate type. These compounds have simple structures with square 2D arrays of metal ions bridged by CN^-^ ligands or lamella that are linked in three-dimensional crystals by pillar ligands, usually pyrazine or diamines. The 2D [M(CN)_4_]^2-^ sheets are grown on a surface functionalized with a SAM with pyridine or amine functional groups. Then, a crystalline layer of the Hoffman clathrate type MOF can be grown using a solution of the components^109,110^ The quMOFs or magnetic MOFs reported so far have more complex structures than simple Hoffman clathrates, but similar techniques could be used for their growth and organization on surfaces.

## Perspectives and challenges

The goal of producing operational molecular devices for spintronics, information storage, or quantum information processing drives countless research efforts. On the one hand, chemists try to prepare molecules with better properties as SMMs or qubits and focus on the characterization of the new species. The milestones achieved include SMMs with hysteresis at 80  K and qubits with relatively long coherence times that allow manipulation for simple quantum algorithms or embedded error correction. The results reported up to 2023 are somehow stagnant on the properties of new materials, thus the field is still open for chemists to contribute to the synthesis of better SMMs and qubits. Additionally, chemistry provides the tools for efficient surface deposition. Large-area deposition of semi-ordered nanomagnets is now possible. A key factor is the design of molecular systems with surface deposition in an ordered manner as one of the required system properties. Many research efforts in chemistry laboratories go in this direction. Physicists, on the other hand, have achieved important milestones in the evaporation of submonolayers or isolated molecules the detection of molecular properties on a surface, and in designing molecular spin quantum processors that could be achieved with current technologies. However, it seems extremely difficult for physicists to move on from their comfort zones and use new materials. Most experiments we see on SMMs or qubits on surfaces are based on the same TbPc and VOPc systems. Now, chemists and physicists alike are studying extremely air and moisture-sensitive metallocenes with ciclooctatetraene ligands^111^ It would be desirable to open the scope of other qubits and SMMs, in particular to the best performers. The main problem is usually evaporation. Most physicists prefer to study molecules that can be evaporated. This limits the list of available materials, and thus the most studied systems are not the best or the most stable. We might see in the coming years with the advances in electrospray deposition that the properties of more molecular SMMs and qubits are studied on surfaces^112–114^ Using electrospray, controlled deposition is now amenable to many more molecules that cannot be evaporated. Very recently, researchers have revisited surface deposition of the classic Fe_4_ and Mn_12_ SMMs using electrospray^115^ Their work unambiguously shows that Mn_12_ and Fe_4_ are intact on the surface, this shows that electrospray can be used as a suitable deposition technique of intact molecules, even for such a complex molecule as Mn_12_. Furthermore, it is necessary to move from studies on single molecules, or small ordered ensembles of isolated molecules to large arrays of molecules, such as monolayers or nanocrystals. This requires controlling the organization of the molecules on a surface. For simple phthalocyanine complexes^116^ this is now well understood but we still lack the control of surface deposition for molecules with more complex structures. It is very challenging to obtain ordered layers or to know the relative orientation of the molecules with respect to each other or the surface. This is one of the very difficult challenges, monolayers or very thin layers can be disordered. In fact this is a great problem in depositing SMMs and qubits, that monolayers of simple molecules already show complicated order-disorder patterns, or ordered patterns with several orientations, even when prepared by evaporation. Surface functionalization can be very useful in this regard, followed by on-surface chemistry between the surface functional group and the molecule. A few examples show that following this route monolayer with only a small number of molecular orientations can be obtained^33,34^ Furthermore, the recent results on electrospray deposition are very promising, and could also allow access to large-area ordered layers.

Proof of principle on isolated complexes that can work as multi-qubits quantum simulators, or that have embedded error-correction show that device integration of molecular nanomagnets is a strong possibility^117^ The advances in magnetic MOFs and quMOFs and in the design and synthesis of magnetic 2D coordination polymers or 2D MOFs hold great promise in the realization of devices based on magnetic molecules. In the last 10 years, researchers have demonstrated that the properties of SMMs on surfaces can be enhanced when compared to the bulk materials in two specific instances: TbPc on MgO/Ag(100)^29^ and a dysprosium metallocene SMM on silica^39^ For qubits, the results obtained so far are much more limited, mainly due to the technical difficulty of measuring T_1_ and T_2_ by EPR on flat surfaces. The introduction of SMMs and qubits in magnetic MOFs or 2D materials offers many possibilities: from controlled surface deposition of flakes to surface growth using surMOFs techniques. 2D materials can be exfoliated in monolayers or flakes of several layers, these flakes can be deposited from solution or using mechanical methods on a surface. For spin crossover materials some groups have already started to explore what they call "straintronics" creating heterostructures with 2D coordination polymers^118^

2D materials offer many possibilities for device integration by mechanical transfer of monolayers to a surface, and even the formation of heterostructures. The area of 2D MOF containing SMMs or qubits has only been recently started to be explored by chemists. The new availability of microED or structural electron diffraction will also come in handy to ascertain molecular orientation on a surface. In a 2D coordination polymer, the relevant qubit or SMM is a node in the polymer. Single crystal structures of nanocrystals can now be elucidated using electron diffraction. This means that 2D coordination polymers or MOFs, that form nanometric crystalline flakes can be structurally characterized unambiguously. Surface deposition of these nanocrystals or flakes leads to a system where the relative qubit/qubit or SMM/SMM orientation respects each other and the surface is known.

We now have a better understanding of some of the factors that so far have precluded the advancement of molecule-based devices exploiting SMMs and molecular qubits, mainly the inability to reproduce or detect the relevant magnetic properties on single molecules, or monolayers on surfaces. For SMMs, this is mainly due to strong electronic interaction between SMM and substrate, while for qubits the electronic interaction with the substrate adds to the lack of an adequate method to characterize their properties on a surface. Improvement in both deposition control and detection of the relevant properties are the two key factors. The next logical step in this roadmap to molecule-based devices for spintronics, information storage and processing is to learn to adequately control these factors to achieve functional molecular devices.

## References

[1] Sessoli, R. et al. High-spin molecules: [Mn_12_O_12_(O_2_CR)_16_(H_2_O)_4_]. *J. Am. Chem. Soc.* **115**, 1804–1816 (1993).

[2] Craig GA, Murrie M (2015). 3D single-ion magnets. Chem. Soc. Rev..

[3] Zabala-Lekuona A, Seco JM, Colacio E (2021). Single-molecule magnets: From Mn12-ac to dysprosium metallocenes, a travel in time. Coord. Chem. Rev..

[4] Woodruff DN, Winpenny REP, Layfield AR (2013). Lanthanide single-molecule magnets. Chem. Rev..

[5] Rosado Piquer L, Sañudo EC, Rosado Piquer L (2015). Heterometallic 3d-4f single-molecule magnets. Dalton Trans..

[6] Guo F (2018). Magnetic hysteresis up to 80 kelvin in a dysprosium metallocene single-molecule magnet. Science (1979).

[7] Sessoli R, Gatteschi D, Caneschi A, Novak MA (1993). Magnetic bistability in a metal-ion cluster. Nature.

[8] Wernsdorfer W, Murugesu M, Christou G (2006). Resonant tunneling in truly axial symmetry Mn 12 single-molecule magnets: Sharp crossover between thermally assisted and pure quantum tunneling. Phys. Rev. Lett..

[9] Ishikawa N, Sugita M, Wernsdorfer W (2005). Quantum tunneling of magnetization in lanthanide single-molecule magnets: bis(phthalocyaninato)terbium and bis(phthalocyaninato)dysprosium anions. Angew. Chem. Int Ed. Engl..

[10] Wernsdorfer W, Bhaduri S, Boskovic C, Christou G, Hendrickson D (2002). Spin-parity dependent tunneling of magnetization in single-molecule magnets. Phys. Rev. B.

[11] Wernsdorfer W, Aliaga-Alcalde N, Hendrickson DN, Christou G (2002). Exchange-biased quantum tunnelling in a supramolecular dimer of single-molecule magnets. Nature.

[12] Brechin EK (2002). Quantum tunneling of magnetization in a new [Mn18]2+ single-molecule magnet with S = 13. J. Am. Chem. Soc..

[13] Leuenberger MN, Loss D (2001). Quantum computing in molecular magnets. Nature.

[14] Stepanenko D, Trif M, Loss D (2008). Quantum computing with molecular magnets. Inorg. Chim. Acta.

[15] Meier F, Levy J, Loss D (2003). Quantum computing with spin cluster qubits. Phys. Rev. Lett..

[16] Lehmann J, Gaita-Ariño A, Coronado E, Loss D (2009). Quantum computing with molecular spin systems. J. Mater. Chem..

[17] Gaita-Ariño A, Luis F, Hill S, Coronado E (2019). Molecular spins for quantum computation. Nat. Chem..

[18] Wasielewski MR (2020). Exploiting chemistry and molecular systems for quantum information science. Nat. Rev. Chem..

[19] Fursina AA, Sinitskii A (2023). Toward molecular spin qubit devices: Integration of magnetic molecules into solid-state devices. ACS Appl. Electron. Mater..

[20] Gabarró-Riera G, Aromí G, Sañudo EC (2023). Magnetic molecules on surfaces: SMMs and beyond. Coord. Chem. Rev..

[21] Barra AL (1999). Single-molecule magnet behavior of a Tetranuclear Iron(III) Complex. The origin of slow magnetic relaxation in Iron(III) clusters. J. Am. Chem. Soc..

[22] Mannini M (2009). Magnetic memory of a single-molecule quantum magnet wired to a gold surface. Nat. Mater..

[23] Lodi Rizzini A (2012). Exchange biasing single molecule magnets: Coupling of TbPc2 to antiferromagnetic layers. Nano Lett..

[24] Lodi Rizzini A (2011). Coupling single molecule magnets to ferromagnetic substrates. Phys. Rev. Lett..

[25] Lodi Rizzini A (2014). Coupling of single, double, and triple-decker metal-phthalocyanine complexes to ferromagnetic and antiferromagnetic substrates. Surf. Sci..

[26] Sanvito S (2010). Molecular spintronics: The rise of spinterface science. Nat. Phys..

[27] Prado Y (2015). Enhancing the magnetic anisotropy of maghemite nanoparticles via the surface coordination of molecular complexes. Nat. Commun..

[28] Rosado Piquer L (2019). Hysteresis enhancement on a hybrid Dy(iii) single molecule magnet/iron oxide nanoparticle system. Inorg. Chem. Front.

[29] Wäckerlin C (2016). Giant hysteresis of single-molecule magnets adsorbed on a nonmagnetic insulator. Adv. Mater..

[30] Rosado Piquer L, Dreiser J, Sañudo EC (2021). Heterometallic Co-Dy SMMs grafted on iron oxide nanoparticles. Dalton Trans..

[31] Piquer LR, Sánchez RR, Sañudo EC, Echeverría J (2018). Understanding the molecule-electrode interface for molecular spintronic devices: A computational and experimental study. Molecules.

[32] Avvisati G (2018). Ferromagnetic and antiferromagnetic coupling of spin molecular interfaces with high thermal stability. Nano Lett..

[33] Gabarró-Riera G, Jover J, Rubio Zuazo J, Bartolomé E, Sañudo EC (2022). Towards large area surface functionalization with luminescent and magnetic lanthanoid complexes. Inorg. Chem. Front.

[34] Tesi L (2023). Modular approach to creating functionalized surface arrays of molecular qubits. Adv. Mater..

[35] Noh K (2023). Template-directed 2D nanopatterning of S = 1/2 molecular spins. Nanoscale Horiz..

[36] Guo FS (2017). A Dysprosium Metallocene single-molecule magnet functioning at the axial limit. Angew. Chem. - Int. Ed..

[37] Goodwin CAP, Ortu F, Reta D, Chilton NF, Mills DP (2017). Molecular magnetic hysteresis at 60 kelvin in dysprosocenium. Nature.

[38] Korzyński MD, Berkson ZJ, Le Guennic B, Cador O, Copéret C (2021). Leveraging surface siloxide electronics to enhance the relaxation properties of a single-molecule magnet. J. Am. Chem. Soc..

[39] Bernhardt M (2023). Tailored Lewis acid sites for high-temperature supported single-molecule magnetism. J. Am. Chem. Soc..

[40] Allouche F (2017). Magnetic memory from Site Isolated Dy(III) on Silicamaterials. ACS Cent. Sci..

[41] Cornia, A. et al. Preparation of Novel Materials Using SMMs. in *Single-Molecule Magnets and Related Phenomena*, 133–161 (Springer Berlin Heidelberg, 2006).

[42] Sessoli R (2017). Magnetic molecules back in the race. Nature.

[43] Coronado E, Yamashita M (2016). Molecular spintronics: the role of coordination chemistry. Dalton Trans..

[44] Sanvito S (2011). Molecular spintronics. Chem. Soc. Rev..

[45] Bogani L, Wernsdorfer W (2008). Molecular spintronics using single-molecule magnets. Nat. Mater..

[46] Margheriti L (2010). X-ray detected magnetic hysteresis of thermally evaporated terbium double-decker oriented films. Adv. Mater..

[47] Gonidec M (2011). Surface Supramolecular organization of a Terbium (III) Double-decker complex on graphite and its single molecule magnet behavior b b. J. Am. Chem. Soc..

[48] Paschke F (2021). Exceptionally high blocking temperature of 17 K in a surface-supported molecular magnet. Adv. Mater..

[49] Borilovic I (2022). Three individually addressable spin qubits in a single molecule. Chem. Commun..

[50] Graham MJ (2014). Influence of electronic spin and spin-orbit coupling on decoherence in mononuclear transition metal complexes. J. Am. Chem. Soc..

[51] Atzori M (2016). Quantum coherence times enhancement in Vanadium(IV)-based potential molecular qubits: the key role of the Vanadyl Moiety. J. Am. Chem. Soc..

[52] Tesi L (2021). Plasmonic metasurface resonators to enhance terahertz magnetic fields for high-frequency electron paramagnetic resonance. Small.

[53] Song L (2016). Toward increased concentration sensitivity for continuous wave EPR investigations of spin-labeled biological macromolecules at high fields. J. Magn. Reson..

[54] Zhang Z-H, Wu S-Y, Xu P, Li L-L (2010). Theoretical studies of the EPR parameters for Ni 2+ and Co + in MgO. Braz. J. Phys..

[55] Thorp JS, Hossain MD (1981). ESR linewidth mechanisms in Ni2/MgO. J. Magn. Magn. Mater..

[56] Baumann S (2015). Electron paramagnetic resonance of individual atoms on a surface. Science (1979).

[57] Chen, Y., Bae, Y. & Heinrich, A. J. Harnessing the Quantum Behavior of Spins on Surfaces. *Adv. Mater.* **35**, 2107534 (2023). 10.1002/adma.202107534 34994026

[58] Wang Y (2023). An atomic-scale multi-qubit platform. Science (1979).

[59] Zhang X (2022). Electron spin resonance of single iron phthalocyanine molecules and role of their non-localized spins in magnetic interactions. Nat. Chem..

[60] Ishikawa N, Sugita M, Ishikawa T, Koshihara S-Y, Kaizu Y (2003). Lanthanide double-decker complexes functioning as magnets at the single-molecular level. J. Am. Chem. Soc..

[61] Kragskow JGC (2023). Spin–phonon coupling and magnetic relaxation in single-molecule magnets. Chem. Soc. Rev..

[62] Layfield R, Guo F-S, Mansikkamaki A, Tong M-L, Chen Y-C (2019). Uranocenium: synthesis, structure and chemical bonding. Angew. Chem. Int. Ed..

[63] Goodwin AP (2017). Synthesis and electronic structures of heavy lanthanide metallocenium cations. J. Am. Chem. Soc..

[64] Jellen MJ, Ayodele MJ, Cantu A, Forbes MDE, Garcia-Garibay MA (2020). 2D arrays of organic qubit candidates embedded into a pillared-paddlewheel metal−organic framework. J. Am. Chem. Soc..

[65] Canarie ER, Jahn SM, Stoll S (2020). disordered, low density packed radicals. J. Phys. Chem. Lett..

[66] Collauto A (2012). A slow relaxing species for molecular spin devices: EPR characterization of static and dynamic magnetic properties of a nitronyl nitroxide radical. J. Mater. Chem..

[67] Schäfter, D. et al. Molecular One- and Two-Qubit Systems with Very Long Coherence Times. *Adv. Mater.* **35**, 2302114 (2023). 10.1002/adma.202302114 37289574

[68] Crivillers N, Mas-Torrent M, Vidal-Gancedo J, Veciana J, Rovira C (2008). Self-assembled monolayers of electroactive polychlorotriphenylmethyl radicals on Au(111). J. Am. Chem. Soc..

[69] de Sousa A (2020). Exploiting the versatile alkyne-based chemistry for expanding the applications of a stable triphenylmethyl organic radical on surfaces †. Chem. Sci..

[70] Zhang L (2016). TEMPO monolayers on Si(100) electrodes: electrostatic effects by the electrolyte and semiconductor space-charge on the electroactivity of a persistent radical. J. Am. Chem. Soc..

[71] Mannini M (2007). Self-assembled organic radicals on Au(111) surfaces: A combined ToF-SIMS, STM, and ESR study. Langmuir.

[72] Poggini L (2021). Engineering Chemisorption of Fe4 single-molecule magnets on gold. Adv. Mater. Interfaces.

[73] Poggini, L. et al. Chemisorption of nitronyl-nitroxide radicals on gold surface: an assessment of morphology, exchange interaction and decoherence time †. Nanoscale **13**, 7613 (2021). 10.1039/d1nr00640a 33881100

[74] Tesi L (2016). Quantum coherence in a processable vanadyl complex: New tools for the search of molecular spin qubits. Chem. Sci..

[75] Malavolti L (2018). Tunable spin-superconductor coupling of spin 1/2 Vanadyl Phthalocyanine molecules. Nano Lett..

[76] Duncan DA (2010). A photoelectron diffraction investigation of vanadyl phthalocyanine on Au (111). Surf. Sci..

[77] Eguchi K, Takagi Y, Nakagawa T, Yokoyama T (2013). Molecular orientation and electronic states of vanadyl phthalocyanine on Si(111) and Ag(111) surfaces. J. Phys. Chem. C..

[78] Adler H (2015). Interface properties of VOPc on Ni(111) and graphene/Ni(111): Orientation-dependent charge transfer. J. Phys. Chem. C..

[79] Cimatti I (2019). Vanadyl phthalocyanines on graphene/SiC(0001): Toward a hybrid architecture for molecular spin qubits. Nanoscale Horiz..

[80] Warner M (2013). Potential for spin-based information processing in a thin-film molecular semiconductor. Nature.

[81] Santanni, F. et al. VdW Mediated Strong Magnetic Exchange Interactions in Chains of Hydrogen-Free Sublimable Molecular Qubits. *JACS Au* **3**, 1250–1262 (2023). 10.1021/jacsau.3c00121 PMC10131211 37124308

[82] Bader K (2014). Room temperature quantum coherence in a potential molecular qubit. Nat. Commun..

[83] Troiani F (2005). Molecular engineering of antiferromagnetic rings for quantum computation. Phys. Rev. Lett..

[84] Ferrando-Soria J (2016). A modular design of molecular qubits to implement universal quantum gates. Nat. Commun..

[85] Larsen, F. K. et al. Synthesis and Characterization of Heterometallic {Cr7 M} Wheels**. Angew. Chem. Int. Ed 42, 101–105 (2003). 10.1002/anie.200390034 19757603

[86] Affronte, M., Carretta, S., Timco, G. a & Winpenny, R. E. P. A ring cycle: studies of heterometallic wheels. *Chem Commun (Camb)* 1789–1797 10.1039/b615543j (2007). 10.1039/b615543j 17476390

[87] Affronte, M. et al. Molecular routes for spin cluster qubits †. *Dalton Trans.,* 2810–2817 10.1039/b515731e (2006). 10.1039/b515731e 16751889

[88] Laye RH (2005). A family of heterometallic wheels containing potentially fourteen hundred siblings. Chem. Commun. (Camb.).

[89] Sañudo, E. C. et al. Al, Ga and In heterometallic wheels and their by-products. *Chem. Commun.* 801–803 (2007). 10.1039/b613877b 17308636

[90] Sañudo EC (2009). Proton NMR study of Cr-Co heterometallic wheel complexes. Inorg. Chem..

[91] Corradini V (2007). Isolated heterometallic Cr7Ni rings grafted on Au(111) surface. Inorg. Chem..

[92] Ghirri, A. et al. Self-assembled monolayer of Cr 7 Ni molecular nanomagnets by sublimation. *ACS Nano* **5**, 7090–7099 (2011). 10.1021/nn201800e 21809833

[93] Corradini V (2012). Magnetic anisotropy of Cr7Ni spin clusters on surfaces. Adv. Funct. Mater..

[94] Bode BE (2023). Dipolar-coupled entangled molecular 4f Qubits. J. Am. Chem. Soc..

[95] Schlimgen AW, Guo Y, Head-Marsden K (2023). Characterizing excited states of a copper-based molecular qubit candidate with correlated electronic structure methods. J. Phys. Chem. A.

[96] Amdur MJ (2022). Chemical control of spin-lattice relaxation to discover a room temperature molecular qubit. Chem. Sci..

[97] Ranieri D (2023). A heterometallic porphyrin dimer as a potential quantum gate: magneto-structural correlations and spin coherence properties. Angew. Chem. - Int. Ed..

[98] Imperato M (2024). Quantum spin coherence and electron spin distribution channels in vanadyl-containing lantern complexes †. Inorg. Chem. Front.

[99] Chiesa A (2023). Blueprint for a molecular-spin quantum processor. Phys. Rev. Appl.

[100] Chen M (2014). Lanthanide-organic coordination frameworks showing new 5-connected network topology and 3D ordered array of single-molecular magnet behavior in the Dy case. Inorg. Chem..

[101] Zhang XJ (2019). Lanthanide chain assembled in metal–organic frameworks: Slow relaxation of the magnetization in Dy(III) and Er(III) complexes. Inorg. Chem. Commun..

[102] Liu, K. et al. Constraining the coordination geometries of lanthanide centers and magnetic building blocks in frameworks: a new strategy for molecular nanomagnets. *Chem. Soc. Rev.* **45**, 2423–2439 (2016). 10.1039/c5cs00770d 27009851

[103] Aulakh D (2015). Metal-organic frameworks as platforms for the controlled nanostructuring of single-molecule magnets. J. Am. Chem. Soc..

[104] Zadrozny JM, Gallagher AT, Harris TD, Freedman DE (2017). A porous array of clock qubits. J. Am. Chem. Soc..

[105] López-Cabrelles J (2021). Near isotropic *D*
_4 *d*_ spin qubits as nodes of a Gd(III)-based metal-organic framework. Inorg. Chem..

[106] Heinke, L., Gliemann, H., Tremouilhac, P. & Wöll, C. SURMOFs: Liquid-phase epitaxy of metal-organic frameworks on surfaces. in *The Chemistry of Metal-Organic Frameworks* 523–550 (John Wiley & Sons, Ltd, 2016). 10.1002/9783527693078.ch17.

[107] Xiao YH, Gu ZG, Zhang J (2020). Surface-coordinated metal-organic framework thin films (SURMOFs) for electrocatalytic applications. Nanoscale.

[108] Liu B (2011). Chemistry of SURMOFs: Layer-selective installation of functional groups and post-synthetic covalent modification probed by fluorescence microscopy. J. Am. Chem. Soc..

[109] Bell CM, Arendt MF, Gomez L, Schmeh RH, Mallouk TE (1994). Growth of Lamellar Hofmann Clathrate films by sequential ligand exchange reactions: assembling a coordination solid one layer at a time. J. Am. Chem. Soc..

[110] Otsubo, K., Haraguchi, T. & Kitagawa, H. Nanoscale crystalline architectures of Hofmann-type metal–organic frameworks. *Coord. Chem. Rev.* **346**, 123–138 (2017).

[111] de Camargo L (2021). Exploring the organometallic route to molecular spin Qubits: The [CpTi(cot)] Case. Angew. Chem. Int. Ed..

[112] Satterley CJ (2007). Electrospray deposition of fullerenes in ultra-high vacuum: In situ scanning tunneling microscopy and photoemission spectroscopy. Nanotechnology.

[113] Saywell A (2011). Single molecule magnets on a gold surface: In situ electrospray deposition, x-ray absorption and photoemission. Nanotechnology.

[114] Rauschenbach S (2009). Electrospray ion beam deposition: Soft-landing and fragmentation of functional molecules at solid surfaces. ACS Nano.

[115] Paschke F, Erler P, Gragnaniello L, Dreiser J, Fonin M (2020). Electrospray deposition and magnetic properties of prototypical molecular magnets. Quantum Mater. Res..

[116] Wang, Y., Wu, K., Kröger, J. & Berndt, R. Review Article: Structures of phthalocyanine molecules on surfaces studied by STM. *AIP Adv.* **2**, 41402 (2012).

[117] Chiesa A, Santini P, Garlatti E, Luis F, Carretta S (2024). Molecular nanomagnets: A viable path toward quantum information processing?. Rep. Prog. Phys..

[118] Boix-Constant C (2022). Strain switching in van der Waals heterostructures triggered by a spin-crossover metal-organic framework. Adv. Mater..

[119] Mannini M (2010). Quantum tunneling of the magnetization in a monolayer of oriented single-molecule magnets. Nature.

[120] Poggini L (2021). Chemisorption of nitronyl-nitroxide radicals on gold surface: An assessment of morphology, exchange interaction and decoherence time. Nanoscale.

[121] Shuter E, Rettig SJ, Orvig C (1995). Oxobis(2,4-pentanedionato)vanadium(IV), a Redetermination. Acta Crystallogr. C..

[122] Ziolo, R. F., Griffiths, C. H. & Troup, J. M. Crystal structure of vanadyl phthalocyanine, phase II. *J. Chem. Soc. Dalton Trans*. 2300–2302 (1980).

[123] Katoh K (2009). Direct observation of lanthanide(III)-phthalocyanine molecules on Au(111) by using scanning tunneling microscopy and scanning tunneling spectroscopy and thin-film field-effect transistor properties of Tb(III)- and Dy(III)-phthalocyanine molecules. J. Am. Chem. Soc..

[124] Hu C, Heger G, Kalf I, Englert U (2005). About the degree of hydration in potassiumtrisoxalatochromate hydrate. Z. fur Kristallogr..

[125] Larsen FK (2003). Synthesis and characterization of heterometallic {Cr_7_M} Wheels. Angew. Chem. Int. Ed..

[126] Habib F (2011). The use of magnetic dilution to elucidate the slow magnetic. J. Am. Chem. Soc..

[127] Urtizberea A (2018). A Porphyrin spin Qubit and its 2D Framework nanosheets. Adv. Funct. Mater..

[128] Urtizberea A (2020). Vanadyl spin qubit 2D arrays and their integration on superconducting resonators. Mater. Horiz..

[129] León-Alcaide L, López-Cabrelles J, Mínguez Espallargas G, Coronado E (2020). 2D magnetic MOFs with micron-lateral size by liquid exfoliation. Chem. Commun..

[130] González J (2021). A multifunctional Dysprosium-Carboxylato 2D metall–organic framework. Angew. Chem. - Int. Ed..

[131] Bartolomé E (2021). Luminescent and magnetic Tb-MOF flakes deposited on silicon. Molecules.

